# Surgical approach towards Grynfelt hernia: A single center experience

**DOI:** 10.1097/MD.0000000000011928

**Published:** 2018-08-17

**Authors:** Kamleshsingh Shadhu, Dadhija Ramlagun, Simeng Chen, Lijia Liu

**Affiliations:** Department of General Surgery, The First Affiliated Hospital of Nanjing Medical University, Nanjing, Jiangsu 210029, PR China.

**Keywords:** lumbar hernia, mesh, open surgery, surgical duration, surgical technique

## Abstract

Due to the rarity of Grynfelt hernia, there is no standardized surgical technique.

From April 2010 to September 2017, 12 cases of primary superior lumbar hernia (Grynfelt hernia) were identified through medical records reviewing and included in our study. Perioperative data, surgical outcomes, and long-term follow-up results were retrospectively analysed.

Male/female ratio of patients was 1:1, with age 69.5 (62, 74.5) [median (25 percentile, 75 percentile)] years old. Right/left side ratio of lumbar hernia was 9/3. The in-hospital duration was 7 (5, 8) days. The surgical duration was 35 (30, 50) minutes. The defect size was 2.1 (1.5, 3) cm in diameter. The discharge of the patient was on 3 (2, 4) post-operative day (POD). The meshes used were: MK5816 (10), MK5810 (1), and UHS (1). There was only 1 case of postoperative complication where the patient was admitted in intensive care unit (ICU) due to hypoxemia. Morbidity, mortality, and recurrence cases were null.

Our study indicates that open surgery with mesh implantation is a reliable way for the management of primary superior lumbar hernia.

## 1 Introduction

A lumbar hernia is the rare protrusion of intraperitoneal or extraperitoneal contents through a defect of the postolateral abdominal wall. They represent <1%–2% of all abdominal wall hernias.^[1]^ It was first coined by Barbette in 1672 and the first case was reported in 1731 by Garangeot. Petit and Grynfelt delineated the boundaries of the inferior and superior lumbar triangles in 1783 and 1866, respectively.^[2]^ Lumbar hernias are either congenital or acquired and the acquired ones are further classified as primary and secondary. The acquired primary lumbar hernias account for 55% of lumbar hernias. Patients with lumbar hernia can present with pathologically flank bulge, accompanying with local discomfort and tenderness.^[3]^ The palpable mass is reducible, which increases in size with increased intra-abdominal pressure and disappears when the patient assumes the prone position.^[4]^ Physical examination plays a very important role in diagnosis. There have been reported cases of lumbar hernias misdiagnosed as lipomas or even gluteal abscess.^[5,6]^ Due to the rarity of this disease, there is lack of specific protocol for its management. Thus, the purpose of our current work is to analyze retrospectively the experience of surgical approach towards this entity in our department.

## 2 Methods

### 2.1 Patients and data collection

Between April 2010 and September 2017, a retrospective single center cohort study was performed. All patients who underwent primary superior lumbar hernia repair at the First Affiliated Hospital of Nanjing Medical University with high expertise in the field of hernia repair were included and followed at the outpatient clinic. All hernias were diagnosed based on clinical examination at the outpatient clinic. If the examination was inconclusive, computed tomography was used to confirm diagnosis. All patients in the study were planned for elective surgical repair. During the study the MK5816, MK5810, and UHS mesh were the treatment of choice in lumbar hernia in our hospital.

The following data were collected using the electronic hospital data system: age, gender, chief complaint, indication of repair, defect size (cm), mesh size (cm), duration of hospital stay (days), duration of surgery (minutes), postoperative complications, number of visits at outpatient clinic, and duration of follow-up. Missing values are reported as unknown. The study passed the review of Ethics Committee of the First Affiliated Hospital of Nanjing Medical University.

### 2.2 Surgical procedure

Surgery was done under general anaesthesia. All patients underwent open approach in the lateral decubitus position. The anatomy of the Grynfelt triangle was made clear; the larger superior triangle is inverted, deeper and more constant. Its boundaries are formed by posterior border of internal oblique (anterior), anterior border of sacrospinalis (posterior), 12th rib and the serratus posterior inferior muscle (base), external oblique and latissimus muscle (roof), and aponeurosis of the transversus abdominis (floor). The latissimus muscle was exposed and opened, content of hernia, normally the retroperitoneal fat was reduced; the defect and lateral side-posterior border of internal oblique, superior side—12th rib and the serratus posterior inferior muscle, medial side- anterior side of sacrospinalis were exposed, preperitoneal (retroperitoneal) space were dissected to exceed the edge of defect for no <3 cm. The defects were repaired using the following mesh (sublay) and the mesh overlapped the defect for at least more than 3 cm: MK5816, MK5810, and UHS. Additional subcutaneous drains were placed if indicated. All patients were asked to the outpatient clinic at a minimum follow-up of 2 months to diagnose recurrence.

### 2.3 Statistical analyses

Statistical analyses were performed with STATA-MP14 (64-bit) Ink. Continuous variables are presented as medians with interquartile range between brackets. Missing values are reported as unknown.

## 3 Results

### 3.1 Patient characteristics

A total of 12 consecutive patients with Grynfelt hernia, 6 males and 6 females, with median age of 69.5 years (62, 74.5) were included in the study. At our center the total number of treated hernias were 4389 for this study time and Grynfelt hernias represented 0.27%. The patients reported no history of other medical conditions or illnesses. The clinicopathologic characteristics of patients are outlined in Table 1.

**Table 1:**
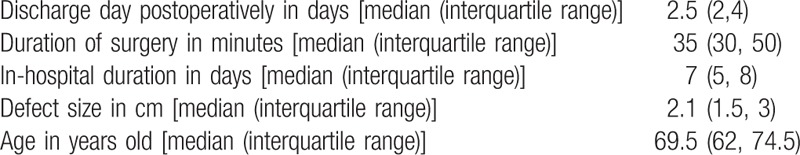
The clinicopathologic characteristics of patients.

### 3.2 Hernia characteristics

Twelve patients were diagnosed with primary superior lumbar hernia out of which nine were right sided. All patients were operated in an elective setting with an open technique under general anaesthesia.

### 3.3 Surgical procedure and hospital stay

All 12 patients underwent open approach in the lateral decubitus position. The mesh used covered the defect for at least more than 3 cm: MK5816 (10), MK5810 (1), and UHS (1). The defects had a median size of 2.1 cm (1.5, 3). The duration of surgeries had a median time of 35 minutes (30, 50). During postoperative period, 11 patients started enteral feeding one day after surgery. One male was admitted to the ICU due to hypoxemia immediately after surgery and was readmitted in the inpatient hernia department after 24 hours of recovery postoperatively. The in-hospital duration was with median days of 7 (5, 8). The discharge time of all 12 patients were of a median of 2.5 postoperative days (2, 4).

### 3.4 Follow-up

All operated-on cases did well on follow-up so far. There was no case of wound infection and mortality in our series. There was no evidence of pain and recurrence of hernia.

## 4 Discussion

**Figure 1:**
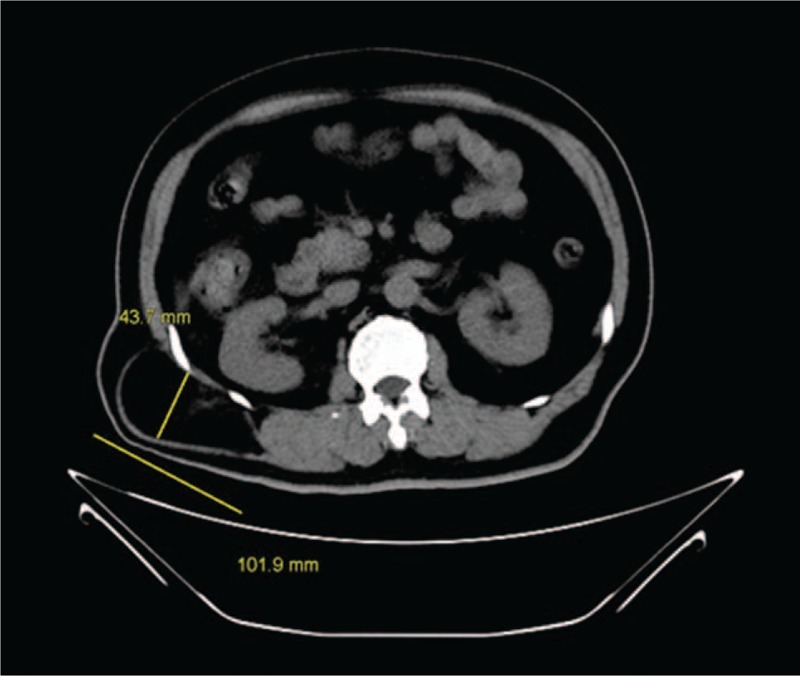
Transverse section of a CT scan imaging showing the primary right lumbar hernia. CT = computed tomography.

**Figure 2:**
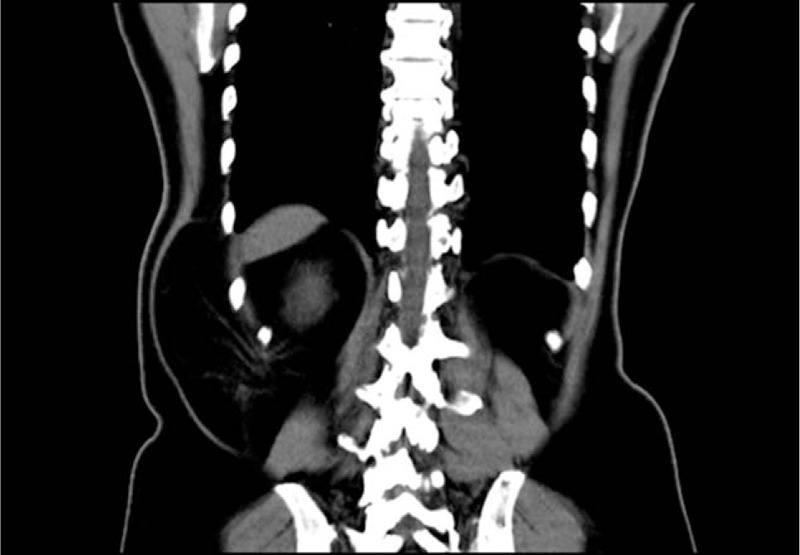
Coronal section of the CT scan showing the right lumbar hernia of the same patient as in Figure 1. CT = computed tomography.

Lumbar hernias are rare and represent < 1%–2% of all abdominal wall hernias. They can occur in the triangle of Grynfelt (superior lumbar hernia) and Petit (inferior lumbar hernia). Grynfelt hernias are the most common^[1]^ and occurred in all patients of our series. CT scan is considered to be the gold standard for the diagnosis and evaluation of the contents of lumbar hernia.^[7]^ Hence, 7 patients in our series had undergone a CT scan imaging for occult hernia or maybe better to evaluate the defect size and hernia contents (Figs. 1 and 2).

Conservative management of lumbar hernias is not suggested because of 2 reasons: around 25% of these hernias are prone to incarceration and 10% to strangulation which may present with features of acute abdomen and need of emergency surgery.^[8]^ Besides, these hernias tend to increase in size with time. Surgical repair of a large lumbar hernia is difficult. Thus, surgery should be performed as early as possible.^[9,10]^ For a high-volume center as ours, open repair was a better choice because it is faster than laparoscopic repair. Based on literature, it has been found that with respect to recurrence rate and morbidity, there was no significant difference between laparoscopic hernioplasty and open hernioplasty.^[11]^

In recent years, many studies have shown that the use of mesh decreases acute pain (up to 12 months) in comparison to tension methods.^[12,13]^ In our series, the mesh covered the edge of the defect for no <3 cm. Fei and Li^[14]^ reported a morbidity of 28% and 0% recurrence for patients with sublay mesh, and in our study the rate of morbidity and recurrence are both 0%.

## 5 Conclusion

After this study of 12 patients, we concluded that surgical repair remains the optimum approach towards this rare abdominal hernia—primary superior lumbar hernia, for our case series. Early diagnosis and management prevents incarceration and strangulation. Long term follow-up is also required for recurrences. However, the limitation of our series is that the number of participants is small and hence, a concrete conclusion can only be drawn based on our experience so far.

## Author contributions

**Conceptualization:** Kamleshsingh Shadhu, Dadhija Ramlagun, Simeng Chen, Lijia Liu.

**Data curation:** Kamleshsingh Shadhu, Dadhija Ramlagun, Simeng Chen, Lijia Liu.

**Formal analysis:** Kamleshsingh Shadhu, Dadhija Ramlagun, Simeng Chen, Lijia Liu.

**Funding acquisition:** Dadhija Ramlagun, Lijia Liu.

**Investigation:** Kamleshsingh Shadhu, Dadhija Ramlagun, Simeng Chen, Lijia Liu.

**Methodology:** Kamleshsingh Shadhu, Dadhija Ramlagun, Simeng Chen, Lijia Liu.

**Project administration:** Kamleshsingh Shadhu, Dadhija Ramlagun, Simeng Chen, Lijia Liu.

**Resources:** Kamleshsingh Shadhu, Dadhija Ramlagun, Lijia Liu.

**Software:** Kamleshsingh Shadhu, Dadhija Ramlagun, Simeng Chen, Lijia Liu.

**Supervision:** Kamleshsingh Shadhu, Dadhija Ramlagun, Simeng Chen, Lijia Liu.

**Validation:** Kamleshsingh Shadhu, Dadhija Ramlagun, Simeng Chen, Lijia Liu.

**Visualization:** Kamleshsingh Shadhu, Dadhija Ramlagun, Simeng Chen, Lijia Liu.

**Writing – original draft:** Kamleshsingh Shadhu, Dadhija Ramlagun, Simeng Chen, Lijia Liu.

**Writing – review & editing:** Kamleshsingh Shadhu, Dadhija Ramlagun, Simeng Chen, Lijia Liu.
